# 

**Published:** 1901-12-21

**Authors:** 


**IMPORTANT NOTICE.**

**On account of the Christmas Holidays, we beg to announce that we shall go to press with our issue of the 28th DECEMBER on MONDAY NIGHT, the 23rd inst. Advertisements intended for insertion in this issue should therefore reach us by 4 p.m. ON MONDAY, the 23rd inst.**

### NOTICE TO CORRESPONDENTS.

All MSS., Letters, Books for Review, and other matters intended for the Editor should be addressed The Editor, "The Hospital," The Hospital Building, 28 & 29 Southampton Street, Strand, W.C.

The Editor cannot undertake to return rejected MS., even when accompanied by stamped directed envelope.

**Notice to Advertisers and Subscribers.**

All Advertisements, Orders for Copies of The Hospital, and Business Communications should be addressed to **THE MANAGER** (**Not to the Editor**), The Hospital, 28 & 29 Southampton Street, Strand, London, W.C.

The Cost of Subscription, post free, is as follows:—Per week, 2½d.; per quarter, 2s. 8d.; per half-year, 5s. 3d.; per annum, 10s. 6d. Foreign:—Per annum, 15s. 2d., payable, in all cases, in advance.

**NOTICE.—Vol. XXX. of "The Hospital" (April 1901, to September 1901), in handsome cloth binding can now be obtained at the office of this Journal, price, 7s. 6d. Cases for binding the Numbers contained in this Volume 1s. 6d. Index to Vol. XXX., 2½d., post free.**

**The Monthly Part for December is now ready. Price 6d., by post 8d.**

### BOOKS RECEIVED.

John Dicks.

"Castles and Abbeys of Great Britain and Ireland."

Charles William Deacon and Co.

"'Ugly,' a Hospital Dog. Told by Himself." By George H. R. Dabbs, M.D.

Charles Griffin and Co.

"Year-Book of the Scientific and Learned Societies of Great Britain and Ireland."

Baillière, Tindall, and Cox.

"The Composition of Dutch Butter." By Dr. J. J. L. van Ryn.

John Leng and Co.

"How to Read, Write, and Debate."

the institution, we are further of opinion that the number of beds will soon prove to be insufficient for such a city as Belfast. The annual cost per head was £24 3s. 4d.

### Scraps and Gleanings.

By a recent resolution of the board of St. Peter's Hospital, Bristol, the special committee appointed in connection with the proposed new infirmary were authorised, on receipt of the formal approval of the Local Government Board to the altered plans, to have the full specifications and particulars of quantities prepared, and to advertise for tenders for the erection of the infirmary The total cost as provided by the final plans is £141,993.

During last year the total number of out-patients who received first aid in the casualty department of the Royal Albert Hospital, Devonport, was 1,484; further treatment was given to 512 of these persons on "free" vouchers, whilst 23 others were treated on payment of a fee of 2s. 6d., and 21 more were offered treatment but declined to pay the fee; 49 others applied for vouchers but were refused as they were considered to be able to pay for surgical advice outside.

### The Student of Medicine.

### APPOINTMENTS.

**Wm. Bannerman,** M.B., C.M.Edin., appointed District Medical Officer of the Clitheroe Union. **Edwin H. Beresford,** M.R.C.S.Eng., L.R.C.P.Lond., appointed Medical Superintendent of New Metropolitan Asylum, Tooting Bec. **Arthur Bevan,** M.B.Lond., M.R.C.S., L.R.C.P.Lond., appointed House Physician to the Hospital for Sick Children, Great Ormond Street. **E. Treacher Collins,** F.R.C.S., appointed Visiting Ophthalmic Surgeon to the Metropolitan Asylums Board Ophthalmic Isolation Schools at Brentwood and Swanley. **John Cunningham,** M.B., C.M.Glasg., appointed Certifying Factory Surgeon for the Stewarton District of Ayrshire. **J. O. Cuthbertson,** M.B., B.Ch.Oxon., appointed Senior House Surgeon, Radcliffe Infirmary, Oxford. **Margaret B. Austin Dobson,** M.B., appointed Assistant Medical Officer to the Bracebridge Asylum, near Lincoln. **Harold Freeth,** M.D.Dub., appointed House Surgeon to the Grimsby and District Hospital. **C. B. Gunn,** M.B.Edin., appointed Certifying Factory Surgeon for the Peebles District. **E. J. F. Hardenberg,** M.R.C.S.Eng., L.R.C.P.Lond., appointed Junior Resident Medical Officer to the North-West London Hospital. **G. Hardwicke,** M.R.C.S.Eng., appointed District Medical Officer of the Newmarket Union. **Miss Jane Buchanan Henderson,** M.D.Brux., L.R.C.P.&S.Edin., appointed Medical Officer to one of the Boer Concentration Camps in the Transvaal. **George A. Johnston,** M.B., Ch.B.Aberd., appointed Assistant Medical Officer at the Sunnyside Asylum, Montrose. **Norman H. Joy,** M.R.C.S.Eng., L.R.C.P.Lond., appointed Medical Officer to the Bradfield Union Workhouse. **T. N. Kelynack,** M.D., M.R.C.P., appointed Assistant Physician to the North London Hospital for Consumption and Diseases of the Chest, Mount Vernon Hampstead, and Fitzroy Square, W. **Lawrie M'Gavin,** F.R.C.S., appointed Clinical Assistant to the Throat Department of Guy's Hospital. **G. Norman Meachen,** M.B., B.S., M.R.C.P., appointed Honorary Physician to the St. Pancras and Northern Dispensary. **Denis Nyhan,** L.R.C.P., L.R.C.S.Edin., appointed Certifying Factory Surgeon for the Brynmawr District of Brecknockshire. **Walter C. C. Parkes,** D.P.H.Camb., F.C.S., appointed Analyst and Bacteriologist to the Transvaal Government. **J. A. Parsons,** M.D., appointed District Medical Officer of the Oakham Union. **G. W. Morris Pritchett,** M.R.C.S.Eng., L.R.C.P.Lond., appointed Senior Resident Medical Officer to the North-West London Hospital. **J. A. Purdon,** L.R.C.S., L.R.C.P.Irel., appointed District Medical Officer of the Spalding Union. **Harry Scholefield,** M.B., B.S.Vict., appointed Second Assistant Medical Officer to the Paddington Parish Infirmary. **W. J. Shannon,** L.R.C.P., L.R.C.S.Edin., appointed District Medical Officer of the Biggleswade Union. **Walter Smartt,** F.R.C.S.I., L.R.C.P.I. L.M., appointed Medical Officer and Public Vaccinator for the Tarporley District of the Tarvin Union. **Stanley Wallace,** M.R.C.S.Eng., L.R.C.P.Lond., appointed Medical Officer of Health of the town of Skegness, Lincs.

Manchester Royal Infirmary.—The following appointments have been made:—House Physician—**W. P. Gowland,** M.R.C.S., L.R.CP. House Surgeons—**H. S. Lister,** M.B., Ch.B.Vict.; **P. H. Green,** M.B., Ch.B.Vict. Assistant Medical Officer—**W. R. Matthews,** M.B., C.M.Aberd. Clinical Assistant at the Convalescent Hospital, Cheadle—**M. B. Arnold,** M.B., Ch.B.Vict

